# Non-Invasive Detection of Extracellular Matrix Metalloproteinase Inducer EMMPRIN, a New Therapeutic Target against Atherosclerosis, Inhibited by Endothelial Nitric Oxide

**DOI:** 10.3390/ijms19103248

**Published:** 2018-10-19

**Authors:** Rafael Ramirez-Carracedo, Laura Tesoro, Ignacio Hernandez, Javier Diez-Mata, Marco Filice, Rocío Toro, Manuel Rodriguez-Piñero, Jose Luis Zamorano, Marta Saura, Carlos Zaragoza

**Affiliations:** 1 Cardiology Department, University Francisco de Vitoria/Hospital Ramón y Cajal Research Unit (IRYCIS), CIBERCV, 28223 Madrid, Spain; rrcarracedo@hotmail.com (R.R.-C.); lauratesoro4@hotmail.com (L.T.); naxete1992@gmail.com (I.H.); jdiezmata@gmail.com (J.D.-M.); 2 Department of Chemistry in Pharmaceutical Sciences, Faculty of Pharmacy, Complutense University (UCM), National Research Centre for Cardiovascular Disease (CNIC), Biomedical Research Networking Center for Respiratory Diseases (CIBERES), 28040 Madrid, Spain; marco.filice1@gmail.com; 3 Cardiology Department, School of Medicine, Cádiz University, 11001 Cadiz, Spain; rociotorogreen@gmail.com (R.T.); manuel.rodriguez.pinero.sspa@juntadeandalucia.es (M.R.-P.); marta.saura@uah.es (M.S.); 4 Cardiology Department, IRYCIS, CIBERCV, 28034 Madrid, Spain; zamorano@secardiologia.es; 5 Biology Systems Department, Physiology, School of Medicine and Health Sciences, Universidad Alcalá (IRYCIS), Alcala de Henares, 28771 Madrid, Spain

**Keywords:** nitric oxide, endothelial nitric oxide synthase, metalloproteinsases, nanoparticles, magnetic resonance imaging, extacellular matrix metalloproteinase inducer EMMPRIN

## Abstract

Lack of endothelial nitric oxide causes endothelial dysfunction and circulating monocyte infiltration, contributing to systemic atheroma plaque formation in arterial territories. Among the different inflammatory products, macrophage-derived foam cells and smooth muscle cells synthesize matrix metalloproteinases (MMPs), playing a pivotal role in early plaque formation and enlargement. We found increased levels of MMP-9 and MMP-13 in human endarterectomies with advanced atherosclerosis, together with significant amounts of extracellular matrix (ECM) metalloproteinase inducer EMMPRIN. To test whether the absence of NO may aggravate atherosclerosis through EMMPRIN activation, double NOS3/apoE knockout (KO) mice expressed high levels of EMMPRIN in carotid plaques, suggesting that targeting extracellular matrix degradation may represent a new mechanism by which endothelial NO prevents atherosclerosis. Based on our previous experience, by using gadolinium-enriched paramagnetic fluorescence micellar nanoparticles conjugated with AP9 (NAP9), an EMMPRIN-specific binding peptide, magnetic resonance sequences allowed non-invasive visualization of carotid EMMPRIN in NOS3/apoE over apoE control mice, in which atheroma plaques were significantly reduced. Taken together, these results point to EMMPRIN as a new therapeutic target of NO-mediated protection against atherosclerosis, and NAP9 as a non-invasive molecular tool to target atherosclerosis.

## 1. Introduction

In the absence of endothelial NO, vascular endothelial cells become dysfunctional, playing a significant role in the pathogenesis of atherosclerosis. Endothelial dysfunction culminates with the loss of endothelial-dependent vessel wall relaxation, increasing leukocyte-endothelial vessel wall adhesion, oxidized Low-density lipoprotein (LDL) uptake, platelet aggregation, pro-inflammatory cytokine expression, and extracellular matrix (ECM) degradation [1,2,3]. However, the mechanisms by which NO prevents atherosclerosis are not fully understood.

The extracellular matrix metalloproteinase inducer (EMMPRIN, CD147, Basigin) plays a pivotal role in the pathogenesis of cardiac and atherothrombotic diseases [4,5,6]. EMMPRIN is a glycoprotein that regulates MMP expression in several cell types, including endothelial cells, vascular smooth muscle cells, monocytes, macrophages and cardiac myocytes. Activation of EMMPRIN requires its glycosylation for a correct protein trafficking to the cell surface, and forming oligomers [7] which bind to its ligand Cyclophilin A (CyPA) [8]. Elevated levels of circulating CyPA are detected under oxidative stress, hypoxia, and inflammation, facilitating the formation of CyPA/EMMPRIN complexes during the onset of acute myocardial infarction [8] and atherosclerosis. The CyPA/EMMPRIN complex leads to platelet activation, adhesion, thrombus formation [9], and foam cell differentiation [10], playing a critical role in plaque progression and vulnerability [11]. Therefore, targeting EMMPRIN may constitute a novel therapeutic approach to promote cardiovascular protection.

Recently, the use of statins, and in particular atorvastatin, was effective in reducing plaque vulnerability at least by downregulating the expression of EMMPRIN [12], while the use of anti-EMMPRIN specific antibodies downsized atheroma plaques, inhibiting the expression of MMPs [13] and monocyte recruitment to the vascular wall [14]. The same also applied in response to acute myocardial infarction, by preserving left ventricular function [5], and disrupting the complex CyPA/EMMPRIN [8]. 

The use of specific antibodies to target proteins, including EMMPRIN, has been investigated [5,8] with uncertain benefits for the patient, for reasons that include absence of therapeutic effect, or even antibody-mediated side effects. Hence, an efficient alternative including smaller binding molecules, may represent a promising approach. In this regard, paramagnetic micelles have been used to target specific proteins present in several pathophysiological conditions, including EMMPRIN, as we did for non-invasive visualization by molecular magnetic resonance imaging (MRI), finding a significant reduction of the left ventricle necrotic area, in murine and porcine models of acute myocardial infarction [15,16].

We previously found that NOS3 prevents the development of atheroma plaques in apoE and NOS3/apoE double KO atherosclerotic mice [1]. However, the role of EMMPRIN in endothelial NO-mediated inhibition of atherosclerosis remains to be elucidated. Here, we used the same paramagnetic micelles for in vivo non-invasive visualization of EMMPRIN in atherosclerotic apoE and NO3/apoE double null mice, to evaluate the hypothesis that targeted inhibition of EMMPRIN may represent a new mechanism elicited by NO against atherosclerosis.

## 2. Results

### 2.1. Human Endarterectomy Samples Express Extracellular Matrix (ECM) Degrading MMP-9, MMP-13 and EMMPRIN

**Figure 1:**
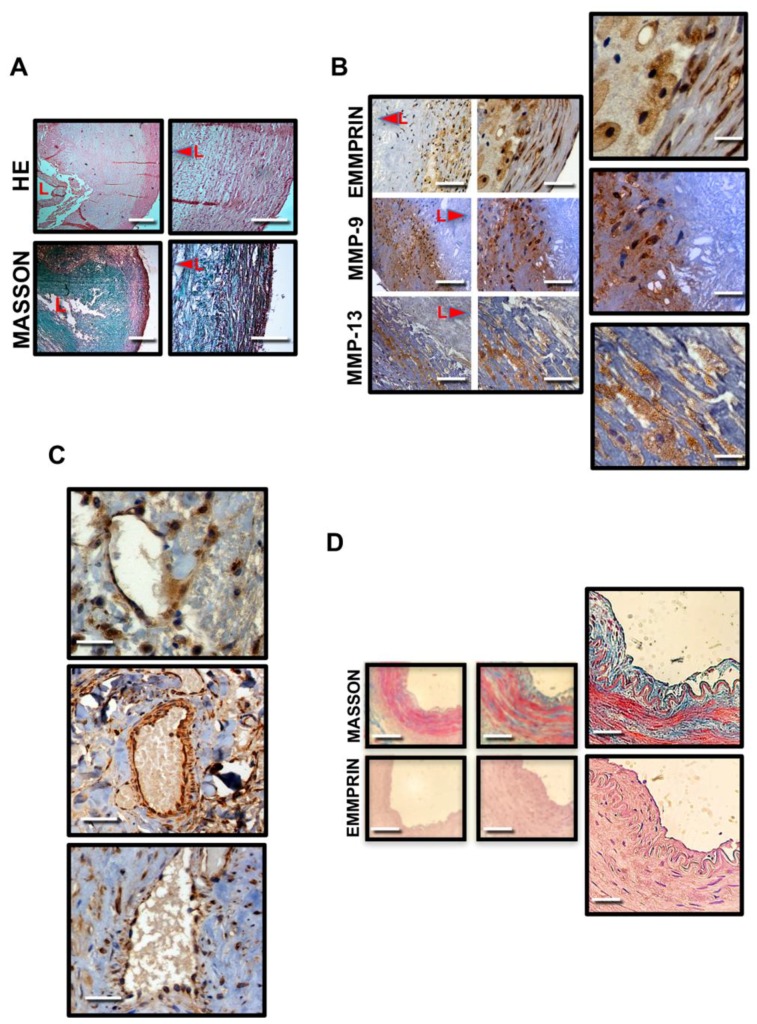
MMP9, MMP-13, and EMMPRIN are expressed in human endarterectomy samples. (**A**) Hematoxylin/Eosin (upper), and Masson Trichrome staining in cross sections of human atherosclerotic carotid arteries. L: lumen. (**B**) Immunostaining of MMP-9, MMP-13, and extracellular matrix metalloproteinase inducer (EMMPRIN) in cross sections of human carotid endarterectomies (Magnifications ×20, ×40 and ×60). (**C**) Representative sections of intraplaque vascularization showing EMMPRIN-positive cells (Magnification ×60). (**D**) Masson Trichrome and EMMPRIN immunostaining of mammary arteries. Scale bars: 50 µm. Arrows point towards the lumen.

The levels of MMP-9, MMP-13, and EMMPRIN were determined by immunohistochemistry in human crossed sections of carotid endarterectomy specimens, showing extensive atherosclerosis, as shown by hematoxylin/eosin, and Masson Trichrome staining (Figure 1A).

Extensive expression of EMMPRIN, MMP-9 and MMP-13 was detected in smooth muscle cells and foam cells, while EMMPRIN was also expressed in endothelial cells of vascularized plaques (Figure 1C), when compared to the levels found in healthy control mammary arteries (Figure 1D), which suggest that EMMPRIN may contribute to the extension of atheroma plaque.

### 2.2. Lack of NOS3 Increases Atherosclerotic Lesions and Inflammatory Macrophages in apoE Null Mice

**Figure 2:**
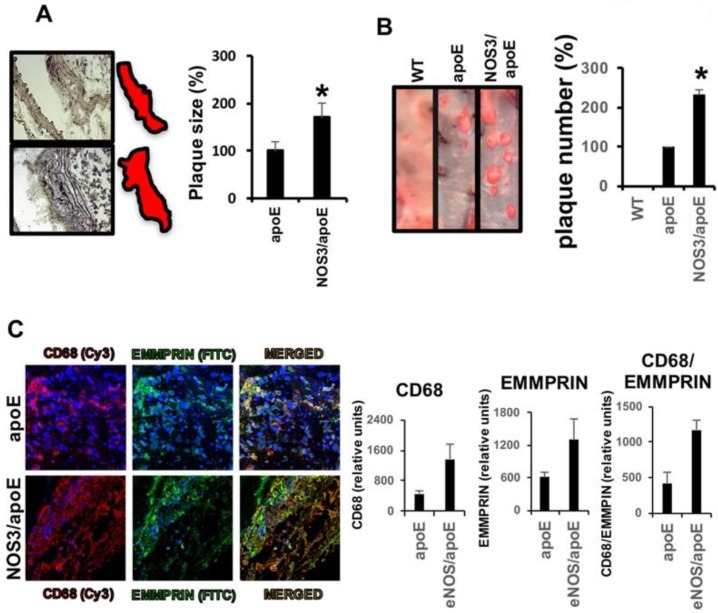
Mice lacking NOS3 show extensive atherosclerotic lesions. (**A**) Hematoxylin/Eosin staining of carotid sections from apoE (upper), and NOS3/apoE null mice (lower), fed with Western-type diet for 12 weeks (*N* = 10 mice/group; apoE: 1671 ± 200 vs. NOS3/apoE: 2834 ± 286; * *P*: 0.002). (**B**) OilRedO staining showing lipid deposition on carotid aortas from the same mice as in (**A**) (*N* = 10 mice/group, apoE: 3.25 ± 1.25 vs. NOS3/apoE: 7.5 ± 1.29; * *P*: 0.003). (**C**) Confocal microscopy detection of CD68 (*N* = 10 mice/group, apoE: 452 ± 77.431 vs. NOS3/apoE: 1352 ± 330. * *P*: 1.27 × 10^−4^; Cy3, red), and EMMPRIN (*N* = 10 mice/group, apoE: 606 ± 91.113 vs. NOS3/apoE: 1308.9 ± 351.364. *P*: 8.85 × 10^−4^; FITC, green) in carotid sections (Nuclei-Hoecht-blue). Merged panels show co-localization of CD68 and EMMPRIN positive cells. (*N* = 10 mice/group, apoE: 410 ± 142.029 vs. NOS3/apoE: 1172 ± 211.784. * *P*: 2.17 × 10^−4^).

ApoE and NOS3/apoE double null mice, when fed for 12 weeks with a Western diet, developed atherosclerotic lesions. Blood glucose content, and total cholesterol and triglyceride content did not show significant differences, while arterial blood pressure was increased in NOS3/apoE null mice, as previously reported [2] (Table 1). Morphometric analysis assayed in hematoxylin/eosin stained carotid sections, and OilRedO staining on full size carotids longitudinally sectioned, revealed that carotid lesion size (apoE: 1671 ± 200 vs. NOS3/apoE: 2834 ± 286; *P*: 0.002), and number (apoE: 3.25 ± 1.25 vs. NOS3/apoE: 7.5 ± 1.29; *P*: 0.003) were significantly increased in NOS3/apoE knockout (KO) mice (Figure 2A,B), suggesting that measurement of NO might correlate with the extension of carotid atherosclerosis. However, since NO is a free radical gas molecule, NO-downstream targets should be used instead for quantification.

We and others found that expression of EMMPRIN is part of an inflammatory response associated with cardiovascular diseases, including abdominal aortic aneurysms [4] and cardiac ischemia/reperfusion injury [5,8]. To test whether EMMPRIN was also expressed in carotid atherosclerotic plaques, we found extensive co-localization of EMMPRIN with CD68 positive infiltrated macrophages in NOS3/apoE double KO mice, when compared to NOS3-expressing animals (Figure 2C), indicating that EMMPRIN is a target of carotid inflammation in atherosclerosis.

### 2.3. NAP9 Targets Extracellular Matrix Metalloproteinase Inducer (EMMPRIN) in Carotid Atherosclerotic Plaques

**Figure 3:**
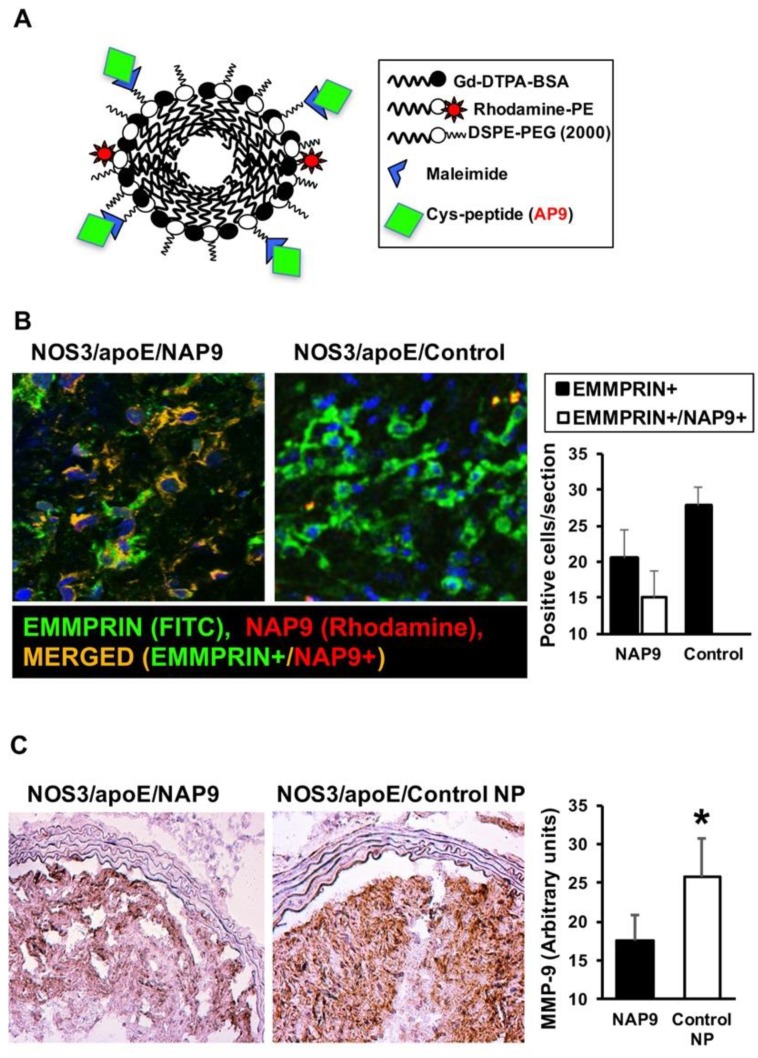
NAP9 nanoparticles (NP) bind to EMMPRIN in carotid atherosclerosis from NOS3/apoE knockout mice. (**A**) Composition of NAP9 micelles containing AP9 peptides. (**B**) Confocal microscopy detection of EMMPRIN (FITC, green) and NAP9 (Rhodamine, red), in carotid sections of NOS3/apoE null mice injected with 0.1 mg/kg/day NAP9 or saline (Control). Merged panels show co-localization of both signals (*N* = 10 mice, mean ± standard deviation (SD)). (**C**) Immunohistochemical detection of MMP-9 in NOS3/apoE double knockout (KO) mice injected with 0.1 mg/kg/day NAP9 or Control NP. (*N* = 10 mice, NAP9: 17.65 ± 3.255 vs. Control NP: 25.73 ± 5.077. * *P*: 0.002).

We previously found that NAP9 nanoparticles, harboring the EMMPRIN-binding peptide AP9 (Figure 3A), specifically target EMMPRIN in acute murine and porcine myocardial infarction [15,16]. To test the potential use of NAP9 in carotid atherosclerosis, mice fed with a Western diet for 12 weeks were intravenously injected with 0.1 mg/Kg/day NAP9 or saline. Carotid sections revealed a strong co-localization between intra-plaque EMMPRIN, as detected by incubation with specific anti-EMMPRIN antibody (FITC, green) with exogenously injected NAP9 (conjugated with Rhodamine, red), as detected by confocal microscopy (merged yellow) (Figure 3B). To further elucidate the contribution of NAP9 in the protection against atherosclerosis, we analyzed the levels of MMP-9, indicative of EMMPRIN activation in carotid arteries. We found that in mice injected with 0.1 mg/Kg/day NAP9, the levels of MMP-9 were significantly reduced, when compared with those found in mice injected with nanoparticle (NP) control (the same nanoparticle lacking AP9: Control NP) (17.57 ± 2.38 vs. 26.26 ± 4.65. Figure 3C). Taken together, our results show NAP9 nanoparticles, may target atherosclerosis in vivo, and reduce the downstream expression of MMP-9. 

### 2.4. Non-Invasive Nanoparticle Detection of EMMPRIN by Magnetic Resonance Imaging (MRI)

**Figure 4:**
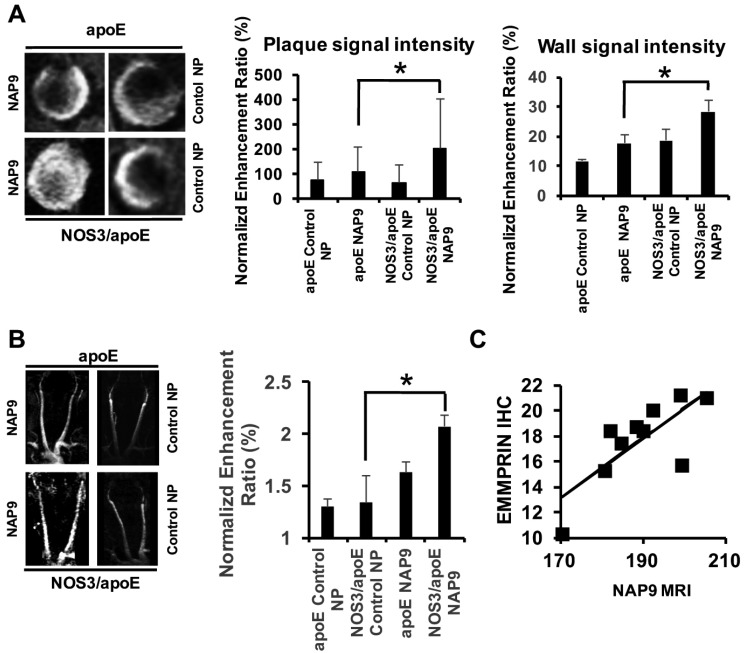
Non-invasive visualization of NAP9 by MRI in atherosclerotic apoE and NOS3/apoE null mice. (**A**) Magnetic resonance image (MRI) of enlarged carotid sections from atherosclerotic apoE and NOS3/apoE null mice injected with 0.1 mg/kg/day NAP9 (left panels) or control (Control NP, right panels). (**Middle**). Carotid plaque signal intensity (*N* = 10 mice/group, apoE/NAP9: 97.704 ± 7.024 vs. NOS3/apoE/NAP9: 189.23 ± 10.380, * *P*: 7.930 × 10^−4^ apoE NAP9 vs. NOS3/apoE NAP9). (**Right**). Carotid vessel wall signal intensity detected by MRI in atherosclerotic apoE and NOS3/apoE injected with 0.1 mg/kg/day NAP9 or Control NP (*N* = 10 mice, apoE/NAP9: 17.57 ± 2.47 vs. NOS3/apoE: 28.44 ± 2.96. * *P*: 2 × 10^−4^). (**B**) Angiography images in the same mice (NOS3/apoE/Control NP: 114.74 ± 27.296 vs. NOS3/apoE/NAP9: 205.16 ± 14.927. * *P*: 3 × 10^−4^). (**C**) Scattered plot representing the extension of NAP9 uptake as detected by MRI (*X* axis) with respect to the amount of EMMPRIN estimated by carotid immunohistochemistry (*Y*-axis) (*R* = 0.757; *P*: 0.011).

**Figure 5:**
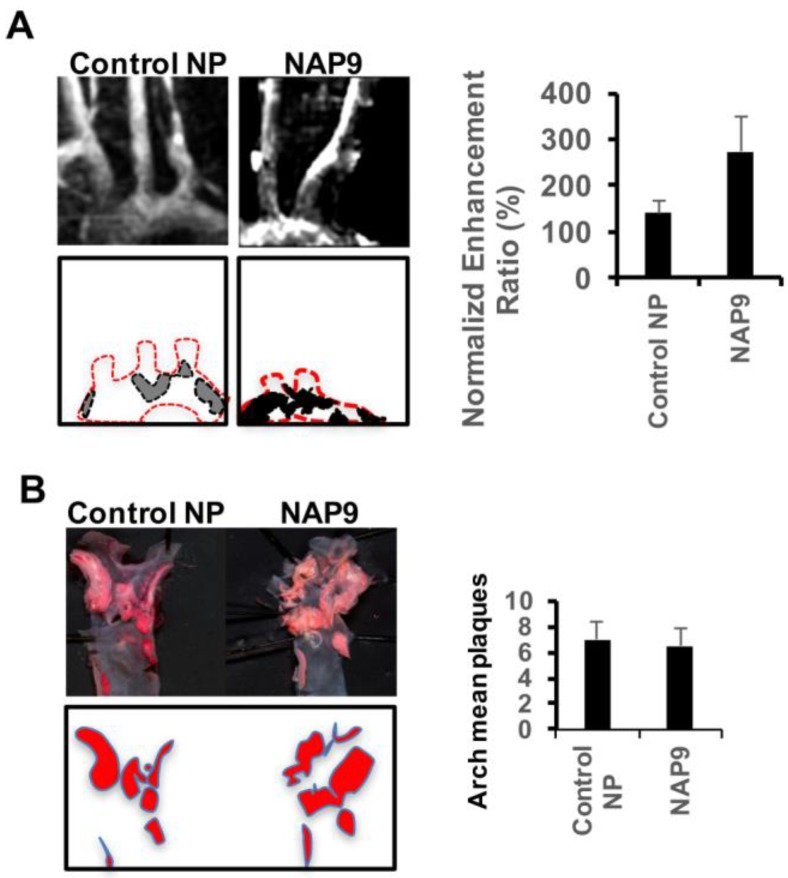
Non-invasive visualization of NAP9 by MRI in the aortic arch of atherosclerotic NOS3/apoE KO mice. (**A**) Images of the aortic arch from NOS3/apoE KO mice injected with 0.1 mg/kg/day control (Control NP, right panel), or NAP9 (left panel). Red dotted lines mark aortic arch and proximal carotid arteries. Black and gray surfaces represent gadolinium enhancement areas corresponding to nanoparticle uptake (Control NP: 139.78 ± 26.288 vs. NAP9: 270.47 ± 73.321; *P*: 1 × 10^−4^). (**B**) OilRedO staining of *en face* aortic arch from the same mice.

We intravenously injected 0.1 mg/kg/day NAP9 or Control NP in apoE and NOS3/apoE KO mice fed with a Western diet for 12 weeks. In accordance with the levels of EMMPRIN ex-vivo detected by confocal microscopy (Figure 3), T1 MRI sequences revealed a gadolinium enhancement in the carotid wall and atheroma plaque of NAP9 injected NOS3/apoE double KO when compared to single apoE null mice (Figure 4A), with similar results detected when carotid angiographies were performed in the same specimens (Figure 4B). A linear regression analysis detected a positive correlation between NAP9 uptake with ex vivo measurement of EMMPRIN in carotid sections from the same mice (Figure 4C; *R* = 0.757, *P*: 0.011), suggesting that NAP9 nanoparticles may represent a new tool for non-invasive targeting of atherosclerosis in vivo. The efficacy of this non-invasive method for plaque detection was also assessed in the aortic arch of NOS3/apoE KO mice intravenously injected with 0.1 mg/kg/day NAP9 or Control NP (Figure 5).

## 3. Discussion

We show the effect of NO in preventing carotid atherosclerosis. Mice lacking NOS3 demonstrate extensive atherosclerotic lesions when compared to NOS3 expressing mice, when fed with high cholesterol diet, both in number and size. Mice lacking NOS3 show increased levels of matrix metalloproteinases MMP-9 and MMP-13 together with extracellular matrix metalloproteinase inducer EMMPRIN, in smooth muscle cells, infiltrated macrophages, and vascular endothelial cells from intraplaque vessels. We used NAP9 nanoparticles to target the expression of EMMPRIN in atherosclerosis, finding specific plaque co-localization in EMMPRIN-expressing cells. Magnetic resonance images taken from mice fed with high cholesterol diet revealed a signal enhancement in the carotids when injected with NAP9 rather Control NP. Non-invasive imaging detection of EMMPRIN, was 2 times increased in the vessel wall, and atheroma plaque, in atherosclerotic NOS3 null mice with respect to single apoE deficient animals, suggesting that NAP9 nanoparticles may represent a new tool for non-invasive in vivo targeting of atherosclerosis. 

We and others found that EMMPRIN is expressed under several inflammatory conditions, including abdominal aortic aneurysms [4], acute myocardial infarction [5,8] and atherosclerosis [1,13]. Besides inflammatory macrophages [10], here we also found that EMMPRIN is present in smooth muscle cells, and endothelial cells from intraplaque vascularization, in human carotid atherosclerosis.

EMMPRIN glycosylation and trafficking from the Golgi to the surface membrane are key steps for EMMPRIN enzymatic activity, in which binding to different proteins is absolutely required. We stated above the role of the complex CyPA/EMMPRIN in the pathogenesis of cardiovascular disease [6]. In addition, EMMPRIN binds to other proteins for proper folding and activation, like MCT-1 and 4 [17], while other complexes regulate EMMPRIN activation itself. Such is the case of Cyclophilin Cyp60, which drives EMMPRIN from the Golgi to the plasma membrane [18]; the binding to the beta-secreatase components, and the relevance in Alzheimer’s disease [19], or the binding with Caveolins, in which the complexes Caveolin-1/EMMPRIN [7], and Caveolin-3/EMMPRIN in the heart [20] prevent EMMPRIN glycosylation, a step required for EMMPRIN self-aggregation, and downstream-mediated expression of MMPs [21,22]. Hence, factors which promote the disruption of certain complexes, may have the power to induce/inhibit EMMPRIN in the context of different pathophysiological conditions. Such is the case of the recent finding in which disruption of the MCT-4/EMMPRIN complex by a small molecule termed Acriflavine, inhibits the hypoxic response, proliferation, and tumor progression in glioblastoma cells [23], or the cardioprotective effect of NO in acute myocardial infarction in which the complex Caveolin-3/low-glycosylated EMMPRIN is stabilized by NO, thus preventing EMMPRIN glycosylation [20].

We previously found that NOS3 prevents aortic atherosclerosis in mice, at least by regulating the expression of MMP-13 [1], by yet unknown mechanisms. Here we show that in the absence of NOS3, the levels of EMMPRIN positive intraplaque macrophages were significant, and correlated with extensive plaque burden, when compared to NOS3 expressing atherosclerotic mice, suggesting that NOS3 may prevent MMP-mediated atherosclerosis through EMMPRIN inhibition, as we evidenced in cardiac myocytes, showing that NO inhibited EMMPRIN mRNA expression by repressing the promoter region [5]. In the absence of NOS3, increased transcriptional expression of EMMPRIN may allow accumulation of high glycosylated forms of EMMPRIN, which help to explain the levels of MMPs in human and murine atherosclerosis. Additionally, the recent findings on NO-mediated EMMPRIN S-nitrosylation may also contribute to inhibit MMP-mediated atherosclerosis [24].

Strategies focused on using EMMPRIN as a therapeutic target against atherosclerosis [8], acute myocardial infarction [5], and different types of cancer [25] have been implemented in preclinical models of disease. During atherosclerosis, EMMPRIN plays a key role in foam cell and plaque formation [9,26], and here we found that NO plays a pivotal role in controlling EMMPRIN-dependent carotid atherosclerosis. The use of antibody-mediated targeting of proteins have been evaluated [27]. However, few human studies were undertaken for reasons that include immune cross reactivity, antibody-mediated side effects, or lack of therapeutic improvement. To avoid undesired side effects, we and others used smaller binding molecules to the target of interest, including specific binding peptides [15,28]. To our knowledge, we were pioneers in using specific EMMPRIN-binding peptides in preclinical animal models of acute myocardial infarction, since they were tested in 293 cells [29], THP-1 cells [30], and in peripheral blood monocyte cell cultures [31].

Paramagnetic micelles have been used to target specific molecules of potential interest, as biomarkers of disease. We bound the specific EMMPRIN binding peptide AP9 [15,16], to gadolinium-based paramagnetic micelles forming nanoparticles (named NAP9), which permitted us to non-invasively visualize EMMPRIN by MRI during the onset and progression of acute myocardial infarction. We targeted here the expression of EMMPRIN in carotid atherosclerosis, providing evidence that the use of non-invasive strategies with theranostic potential, narrowing specific molecules involved in atherosclerosis like EMMPRIN, may have a significant impact when it comes to future improvement in the precision of diagnostic and management of coronary artery diseases, including coronary atheroma plaque vulnerability or acute coronary syndrome, with promising implications in the future of clinical practice focused on the prevention, diagnostic and follow up progression of disease.

In conclusion we have pointed here a new mechanism by which endothelial NO prevents carotid atherosclerosis, through inhibition of EMMPRIN-mediated MMP formation in macrophages, smooth muscle cells, and endothelial cells from intraplaque vessels, providing a new therapeutic tool for non-invasive visualization and targeting of plaque formation and development. Limitations of the study may include the use of male younger mammary arteries as controls in the human analysis of carotid endarterectomies, and the animal model itself, since human and mouse arteries and atheroma plaque composition differ from each other. Therefore, further studies in porcine models of carotid atherosclerosis will be crucial for future testing in humans.

## 4. Materials and Methods

### 4.1. Reagents

Histological reagents, and secondary antibodies were from Sigma (St. Louis, MO, USA). DAB substrate was from Dako (Carpinteria, CA, USA). ECL was from GE Life Sciences (Barcelona, Spain). Anti-MMP-13 (sc-101564), anti-MMP-9 (sc-13520), anti-EMMPRIN (sc-71038), and anti-CD68 (sc-20060) primary antibodies were from Santa Cruz Biotechnology (Santa Cruz, CA, USA). Nanoparticle components were form Avanti Polar Lipids (LabClinics, Barcelona, Spain).

### 4.2. Human Arterial Specimens

The research was performed according to the rules of the Declaration of Helsinki of 1975, revised in 2008. The study was approved by the ethics committee from the University Francisco de Vitoria, Spain, and written consent was achieved from the subjects of the study. Angiography and Doppler ultrasound were used to select carotid endarterectomies from 24 patients (16 men: mean age, 70 ± 5 years; 8 women: mean age, 75 ± 8 years). Mammary arteries were obtained as surgical residues from 10 male patients (mean age, 60 ± 4 years), and used as healthy controls. 

### 4.3. Animals and Diet

Animal research was performed according to the Guide for the Care and Use of Laboratory Animals (US National Institutes of Health, NIH Publication No. 85-23, and revised 1996).

NOS3 and apoE knockout animals were acquired from The Jackson Laboratory (Bar Harbor, ME, USA), to generate the NOS3/apoE double knockout genotype. Four-week-old male mice were fed with Western diet (Harland Tekla, TD88137) for 12 weeks; 16-week-old mice were used for experimentation. 

### 4.4. Blood Lipids and Glucose

After overnight fasting, blood samples were taken fom mice fed with Western diet, and total cholestrol, triglicerides, and glucose levels were measured at the biochemistry facilities of the hospital.

### 4.5. Blood Pressure

Arterial blood pressure was measured in concious mice prewarmed to 30 °C, using a tail-cuff sphyngomanometer (LE 5001; Letica scientific instruments). To prevent stress animals were trained for 5 consecutive days before measurements.

### 4.6. Quantitation of Lesion

Lesions were quantited by carotid staining with OilRedO as previously described [1]. In brief, carotids were longitudinally sliced, incubated with OilRedO solution for 30 min, and washed 3 times with saline buffer to remove unbound reagent. Carotids were scanned and the percentages of plaque area and plaque number were analyzed in a double blinded fashion by using ImageJ, image analysis software (National Institutes of Health, Bethesda, MA, USA).

### 4.7. Confocal Microscopy

Confocal microscopy was performed with specific anti-EMMPRIN, and anti-CD68, primary antibodies, as described [15]. Paraffin embedded carotid sections were incubated with primary antibodies, washed with PBS buffer and incubated with the appropriated secondary antibodies. Sections were mounted with Hoechst containing media and evaluated in a SP5 Leica confocal microscopy.

### 4.8. Peptide and Nanoprobe Composition

Amino acid sequence of AP9 peptide: YKLPGHHHHYRP. 

Paramagnetic nanoparticles were synthesized by the lipid film hydration procedure, as described [15]. In brief, rotary evaporation of Rhodamine-PE, Gd-DTPA-bis (GdDTPA-BSA), 1,2-distearoyl-sn-glycero-3-phosphoethanolamine-N-[methoxy (polyethylene glycol)-2000] (DSPE-PEG2000), and DSPE-PEG2000-maleimide, were used to generate a lipid film in a molar ratio o 10:50:39:1. Peptide AP9 was conjugated with maleimide in a molar ratio micelle:peptide of 1:40. High-performance liquid chromatography (HPLC) was used to estimate the number of AP9 conjugated per nanoparticles (4 ± 2).

### 4.9. Magnetic Resonance

Magnetic resonance (MR) assays were developed on a 4.7 Tesla Bruker Biospec 47/40 (Bruker Biospin, Ettlingen, Germany) with a 6 cm gradient, achieving a top gradient of 450 mT/m as described [4]. Axial, sagittal and coronal images were acquired by using T1 weighted spin echo sequences. After that, respiratory gated T1-weighted spin echo images were taken with 700 ms repetition time. Angiography was performed by using gradient echo, including flow compensation imaging sequences with acquisition matrix size of 256 × 128, FOV 3.5 × 1.75 cm^2^, including 64 slices. Data was interpolated to obtain matrix of 256/128/64 with a FOV of 3.5/1.75/2.5 cm^3^. MIP algorithms were used to the data to generate a 3 dimensional view.

Signal to noise ratio (SNR), of a particular region of interest (ROI) was defined as follows:

$$\;\mathrm{NER}\;\left(\%\right)=\frac{\left[\frac{\mathrm{SNRca}}{\mathrm{SNRt}}\right]\mathrm{Post}\;\mathrm{injection}-\left[\frac{\mathrm{SNRca}}{\mathrm{SNRt}}\right]\mathrm{Pre}\;\mathrm{injection}}{\left[\frac{\mathrm{SNRca}}{\mathrm{SNRt}}\right]\mathrm{Pre}\;\mathrm{injection}}\;$$

### 4.10. Statistical Analysis

Unless otherwise specified, data were presented as means ± SD. Statistical significance was determined by Student’s *t* test (two side unpaired). Differences were considered statistically significant at *P* < 0.05. 

## Figures and Tables

**Table 1:** Fasting blood glucose, lipid content and blood pressure.

Parameters	apoE	NOS3/apoE
Systolic BP (mmHg)	122.1 ± 7.33	141 ± 5.03 (*P*: 0.003)
Diastolic BP (mmHg)	82.1 ± 6.33	106 ± 9.08 (*P*: 0.003)
Blood glucose (mg/dL)	115.6 ± 10.40	121.3 ± 13.86
Total Cholesterol (mg/dL)	811.1 ± 132	821.6 ± 128.82
Triglicerides (mg/dL)	174.7 ± 20.56	171.5 ± 22.07
